# Withaferin A and Celastrol Overwhelm Proteostasis

**DOI:** 10.3390/ijms25010367

**Published:** 2023-12-27

**Authors:** Nuria Vilaboa, Richard Voellmy

**Affiliations:** 1 Hospital Universitario La Paz-IdiPAZ, 28046 Madrid, Spain; 2 CIBER de Bioingenieria, Biomateriales y Nanomedicina, CIBER-BBN, 28046 Madrid, Spain; 3 HSF Pharmaceuticals SA, 1814 La Tour-de-Peilz, Switzerland

**Keywords:** proteostasis, protein unfolding, protein aggregation, Withaferin A, celastrol, IHSF

## Abstract

Withaferin A (WA) and celastrol (CEL) are major bioactive components of plants that have been widely employed in traditional medicine. The pleiotropic activities of plant preparations and the isolated compounds in vitro and in vivo have been documented in hundreds of studies. Both WA and CEL were shown to have anticancer activity. Although WA and CEL belong to different chemical classes, our synthesis of the available information suggests that the compounds share basic mechanisms of action. Both WA and CEL bind covalently to numerous proteins, causing the partial unfolding of some of these proteins and of many bystander proteins. The resulting proteotoxic stress, when excessive, leads to cell death. Both WA and CEL trigger the activation of the unfolded protein response (UPR) which, if the proteotoxic stress persists, results in apoptosis mediated by the PERK/eIF-2/ATF4/CHOP pathway or another UPR-dependent pathway. Other mechanisms of cell death may play contributory or even dominant roles depending on cell type. As shown in a proteomic study with WA, the compounds appear to function largely as electrophilic reactants, indiscriminately modifying reachable nucleophilic amino acid side chains of proteins. However, a remarkable degree of target specificity is imparted by the cellular context.

## 1. Introduction

**Figure 1:**
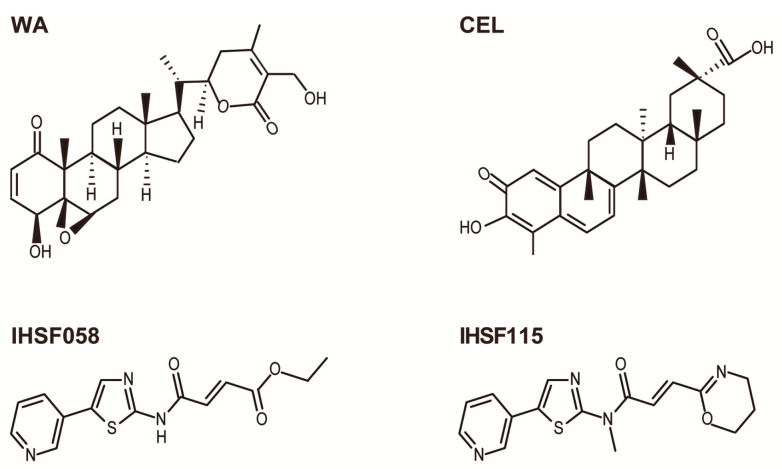
Structures of compounds.

Withaferin A (WA) is abundantly present in the roots and leaves of *Withania somnifera*, a plant that grows in India, the Middle East, and parts of Africa [1,2]. The plant has been used for ages in Ayurvedic medicine for the treatment of a wide variety of medical conditions. The plant is commonly known under the name of Ashwagandha. WA is a steroidal lactone. It contains an α,β-unsaturated carbonyl group in ring A and an epoxy group in ring B (Figure 1). These electrophilic groups are capable of and have been shown to react with nucleophilic side chains of amino acids (mainly cysteine) in proteins.

WA has been reported to possess a variety of therapeutic properties, including antioxidant, anti-inflammatory, antibacterial, antistress, antidiabetic, antipyretic, cardioprotective, neuroprotective, and anticancer activities [3,4,5,6,7,8,9]. The possibility of using WA as a broadly active cancer therapeutic has attracted particular attention and over the years has resulted in a multitude of studies employing various in vitro and in vivo cancer models (reviewed in, e.g., in refs. [1,2]). These studies have identified a large number of proteins and pathways, listed in Table 1 ([4,10,11,12,13,14,15,16,17,18,19,20,21,22,23,24,25,26,27,28,29,30,31,32,33,34,35,36]; adapted from ref. [1]), that are affected by WA in different cell types.

*Tripterygium wilfordii* Hook F is a perennial vine that grows in southeast and southern China. The plant has been used widely in traditional Chinese medicine and is also known as “Thunder of God vine” or “Seven-step vine” [37,38]. Extracts of root bark or mashed bark, contemporaneously formulated as tablets, have been used for treating rheumatoid arthritis, systemic lupus erythematosus, ankylosing spondylitis, Crohn’s disease, psoriasis, nephropathy, neurodegenerative diseases, male fertility regulation, human kidney allograft rejection, and various cancers [39,40,41,42,43,44,45,46,47,48,49,50,51]. The plants contain hundreds of bioactive molecules, the most abundant of which is celastrol (CEL). CEL is a quinone methide triterpenoid and, therefore, contains an α,β-unsaturated carbonyl group (Figure 1). The compound has been reported to possess anti-inflammatory, antioxidant, anticancer, neuroprotective, anti-Gaucher disease, cardioprotective, anti-thrombotic, anti-osteoarthritic anti-allergic, anti-obesity, anti-depressant, anti-liver fibrosis, anti-microbial, anti-fungal, anti-Alzheimer’s disease, anti-systemic lupus erythematosus, and anti-angiogenic activities [52,53,54,55,56,57,58,59,60,61,62,63,64,65,66,67,68,69,70,71,72,73,74,75,76,77,78,79,80]. There has been great interest in the potential of CEL as an anticancer drug. A multitude of studies identified proteins and pathways that are affected by CEL in various types of cancer cells. A short list of some of these findings is presented in Table 2 (information taken from ref. [37]).

Hence, both WA and CEL target, directly or indirectly, a multitude of proteins and pathways. These pleiotropic activities of the compounds suggest that they function more as reactants than as drugs that interact with specific proteins or affect a particular process. This is further supported by the finding that WA and CEL share a number of common targets although the structures of the two compounds are not closely related. Both compounds are reported to cause an increase in reactive oxygen species (ROS), inhibit the proteasome system and autophagy, and inhibit nuclear factor ‘kappa-light-chain-enhancer’ of activated B-cells (NF-kB), signal transducer and activator of transcription 3 (STAT3), and heat shock protein 90 (HSP90), as well as affect the activities of AKT, mammalian target of rapamycin (mTOR), ribosomal protein S6 kinase (p70S6K), 5’ AMP-activated protein kinase (AMPK), and tumor protein p53 (p53) (see Table 1 and Table 2).

Two compounds of the IHSF series of synthetic compounds, IHSF115 and IHSF058, are included in our discussion. These compounds were previously described as inhibitors of heat shock factor 1 (HSF1) and proteostasis interrupters, respectively [119,120]. The compounds comprise an α,β-unsaturated carbonyl group but share no other structural motif with either WA or CEL (Figure 1).

The activities of WA and IHSF are largely neutralized by the strong nucleophile N-acetylcysteine (NAC) [120,121]. This suggests that the electrophilic groups of the compounds are responsible for their activities, including their cytotoxicity, which electrophilic groups covalently modify proteins at cysteines, and possibly other nucleophilic side chains of amino acids. Reduction of the α,β-unsaturated carbonyl groups of WA and IHSF also inactivates the compounds. Reduction of the quinone methide group of CEL has more complex effects. Some activities are abolished [53,107,120], whereas others persist [38]. The covalent modification of proteins by CEL but not by IHSF is reversible (ref. [122] and our unpublished observations).

As discussed above, many targets of WA and CEL have been identified by individual studies that focused on particular effects of WA or CEL that could be demonstrated in the cell lines studied. For many targets, it remained unknown whether they interacted directly with the compounds or whether their activity changed as a consequence of the direct interaction of the compounds with an upstream or interacting target. A better understanding of the sequence of events triggered by the compounds could be expected from systematic studies aimed at discovering the direct targets of the compounds, i.e., the proteins that are covalently modified by them.

## 2. Proteomic Studies of WA Action

### 2.1. Direct Targets of WA and Effects on Protein Expression

Proteomic studies were performed to systematically identify direct targets of WA or to discover proteins that are up- or downregulated in WA-exposed cells. In these studies, cellular proteins were stable-isotope-labeled with heavy or light amino acids [123,124] or a specific probe (biotinylated WA) was used or both approaches were combined [124]. Protein identity was determined with mass spectrometry. One of these studies employed a mouse microglial cell line [123] and the other a human multiple myeloma cell line [124]. The earlier study identified four downregulated proteins that may be either direct or indirect targets of WA [123]. The second study discovered 53 proteins that are downregulated in WA-treated cells and 209 direct targets of WA [124]. The proteins identified in the two studies are compiled in Table S1.

Before going on, it may be worthwhile to discuss a property of WA that is shared with CEL and that may seem unusual but can be readily rationalized. The compounds appear to have beneficial effects at low concentrations (or perhaps also early during exposure of cells to elevated concentrations) but are cytotoxic at elevated concentrations. For example, CEL was found to have general cytoprotective and neuroprotective activities at low concentrations [56,125]. These activities may be explained, at least in part, by the observed activation of HSF1 and the consequential overexpression of genes transactivated by HSF1 that include the genes for the classical heat shock proteins (HSPs). HSPs are the primary protein chaperones that assist in protein folding and the refolding of non-native proteins. WA was also found to stimulate the expression of HSPs at low concentrations [121]. At higher concentrations, both WA and CEL inhibit HSF1 function [120]. As will be elaborated below, exposure to WA causes the pervasive (partial) unfolding of cellular proteins. Increased levels of non-native proteins trigger the activation of HSF1, resulting in HSP overexpression. When the level of non-native proteins does not exceed the capacity of the cells to dispose of these proteins, the cells will recover. The elevated levels of HSPs will persist for some time, during which the cells will be capable of effectively coping with another proteotoxic onslaught. The general phenomenon of induced extended protection is well known and is referred to as “preconditioning”. Analogously, and this may also contribute to the observed cytoprotective effects, WA-mediated inactivation of stress sensor Kelch-like ECH-associated protein 1 (KEAP1) may result in the activation of nuclear factor erythroid 2-related factor 2 (NRF2) that controls a key antioxidative pathway at low compound concentrations. The pathway may be disabled at elevated compound concentrations owing to the inactivation of some of the induced antioxidative proteins [126]. CEL was also found to activate the NRF2 pathway [53]. Other stress-related pathways, such as, e.g., the unfolded protein response (UPR), may be similarly protective at low compound concentrations but may be overwhelmed at elevated concentrations, promoting cell death.

Dom et al. [126] attempted to synthesize the findings of the above proteomic studies, also taking into account results from earlier studies that focused on particular targets of WA [127,128]. The authors focused on several major subsystems of the proteostasis network and pathways. The pathways considered and the proteins directly targeted or downregulated by WA deemed relevant are listed in Table 3. To allow the reader to understand why particular proteins were selected for discussion, we have included in Table 3 the authors’ explanations of the functions of the proteins. As mentioned before, the regulator of the NRF2-controlled oxidative stress pathway KEAP1 was found to be a direct WA target. If the binding of WA inactivated KEAP1, this would result in an elevated nuclear concentration of NRF2 and the activation of the expression of the many regulated genes [129]. However, several antioxidative enzymes, including peroxiredoxin 1, glutathione peroxidase 1, and phospholipid hydroperoxide glutathione peroxidase, are also targeted or downregulated by WA, which may weaken or negate antioxidative responses.

WA may also reduce proteasome-mediated protein degradation. Proteasome subunit beta type-5, a subunit of the 20S proteasome that displays a chymotrypsin-like activity, had previously been reported to be covalently modified by WA [130]. In addition, two ubiquitin carboxyl-terminal hydrolases were WA-modified. Autophagy may also be affected by WA. Most notably, histone deacetylase 6 is a direct WA target. This protein plays a critical role by directing ubiquitinated proteins to the autophagy pathway. Several other proteins that are involved in autophagy were found to be targeted or downregulated by WA (phospholipase A-2-activating protein, WD repeat domain phosphoinositide-interacting protein 2, SNARE-associated protein Snapin, the beta chain of tubulin and annexin A4). Multiple proteins involved in ER-associated protein degradation are also modified by WA.

WA also compromises the heat shock response. HSF1, the main transcription factor controlling the response, and the HSP90 proteins, key components of the response, are among the direct WA targets. In addition, two chaperone DNAJ homologs, an HSP70 protein, and HSP70 co-chaperone BAG2 are either covalently modified or downregulated.

Isomerase and disulfide reductase functions which are important for the correct folding of proteins may also be hampered by WA. Peptidyl-prolyl cis–trans isomerase D and several other peptidyl-prolyl isomerases, as well as disulfide reductase DnaJ homolog subfamily C member 10, are covalently modified by WA.

Dom et al. [126] also highlighted numerous WA-targeted proteins associated with kinase functions, cytoskeleton function, protein translation, and the cell cycle. Hence, any or all of the latter processes may be negatively affected by WA. Dysregulation of the NF-kB pathway is believed to be an important aspect of many cancers. The tumor-inhibiting effects of WA had previously been attributed to the inhibition of the kinases (IKK) that phosphorylate and inactivate IkB, the inhibitor of NF-kB, preventing the activation of transcription factor NF-kB [131,132]. Several additional proteins that are involved in NF-kB signaling were identified as direct targets of WA, including the p65 and p50 subunits of NF-kB, coiled-coil domain-containing protein 22 that promotes ubiquitination of IkB, activating signal co-integrator 1 complex subunit 2 that transactivates NF-kB expression, ELKS/Rab6-interacting/CAST family member 1, a regulatory subunit of the IKK complex, and COMM domain-containing protein 3 that downmodulates NF-kB activation.

Regarding downregulated proteins, an observed decrease in the level of a protein may relate to a reduced rate of transcription or translation, to an increased rate of elimination or sequestration (aggregation) as a result of its covalent modification by WA, or to the dissociation of a stabilizing protein complex subsequent to the modification of a partner protein by WA. Hence, the downregulation of proteins may not be very helpful for defining the immediate activity of WA, although it may importantly affect ultimate outcomes.

In summary, the findings discussed suggest, or at least suggest the possibility, that covalent modification by WA or WA-mediated downregulation of components of various pathways or systems such as oxidative stress responses, the heat shock response, the proteasome, autophagy or protein isomerization functions, protein translation, the cell cycle, or NF-kB signaling may explain how WA-mediated cell death is triggered. However, firm conclusions cannot be reached for several reasons. For a majority of direct WA targets, it remains unknown whether the binding of WA in fact inhibits their activity or function. Furthermore, for any downregulated protein, it is unknown whether it can be downregulated to an extent that is sufficient to result in a biological effect. Finally, it is unknown whether cell death is caused by the parallel disablement of multiple pathways or systems or whether the inhibition of a particular pathway or system dominates. Answers may well be cell-type-specific.

### 2.2. Protein Unfolding Induced by WA

**Figure 2:**
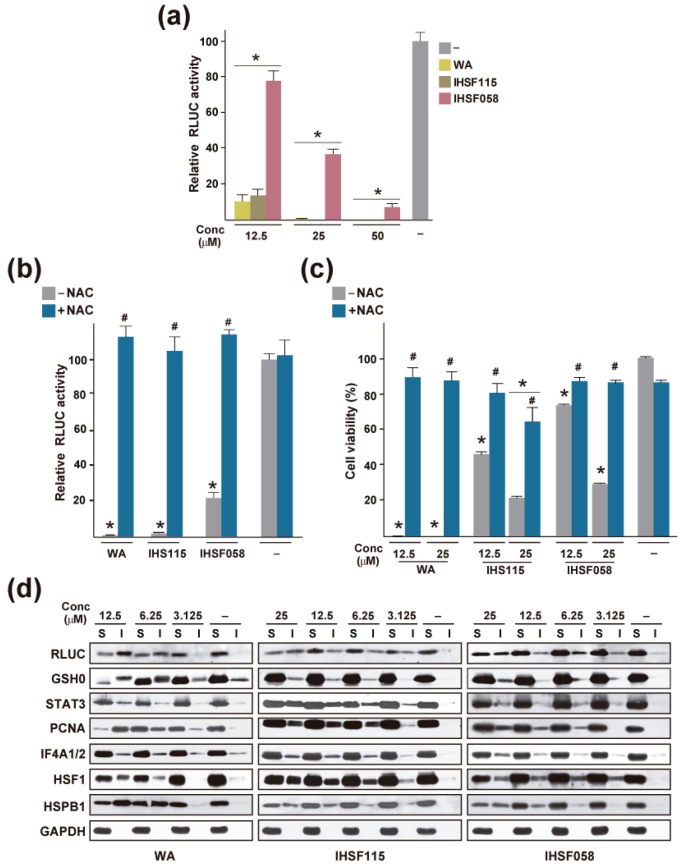
Protein unfolding induced by WA, IHSF115, and IHSF058. (**a**) Inactivation of RLUC in M1 cells (HeLa cells stably transfected with a constitutively expressed RLUC gene) exposed for 6 h to vehicle (−) or compounds at the indicated concentrations. *: *p* ≤ 0.05 (compared to cells exposed to vehicle). (**b**) RLUC activities in M1 cells exposed for 6 h to compounds at 25 µM in the absence or presence of 15 mM NAC. *: *p* < 0.05 (compared to cells exposed to vehicle). #: *p* < 0.05 (compared to cells treated with a compound in the absence of NAC). (**c**) Viability of HeLa cells exposed for 96 h to the indicated concentrations of compounds in the presence or absence of 5 mM NAC. Viability was estimated with an alamar blue assay. *: *p* < 0.05 (compared to cells exposed to vehicle). #: *p* < 0.05 (compared to cells treated with the same dose of compound in the presence of NAC). (**d**) Western blots documenting the unfolding of selected proteins in M1 cells exposed for 6 h to compounds at the indicated concentrations. S: detergent-soluble extract fraction; I: detergent-insoluble extract fraction. The data shown were taken from ref. [120].

A recent study by Vilaboa et al. [120] employed a luciferase inactivation assay as a general assay for tracking induced protein unfolding. It was observed that a 6 h exposure of HeLa-derived cells expressing a Renilla luciferase (RLUC) reporter gene to WA and other compounds such as compounds of the IHSF series HSF115 and IHSF058 resulted in a substantial reduction in luciferase activity (Figure 2a). As demonstrated for IHSF115, this finding could be observed in a variety of different cell lines [120]. Co-exposure to the nucleophile NAC, which was expected to neutralize the electrophilic groups of the compounds, i.e., the α,β-unsaturated carbonyl and epoxy groups, prevented the inactivation of RLUC, suggesting that the observed inactivation was a result of covalent modification of RLUC by the compounds (Figure 2b). Exposure of the cells to WA, IHSF115, or IHSF058 also reduced viability dramatically (at the elevated concentrations of compounds employed), and loss of viability was prevented by co-exposure to NAC (Figure 2c). This finding suggested that the covalent modification of proteins resulted in cell death. At exposure times of up to 6 h, inhibition of proteasome activity did not affect the degree of RLUC inactivation [120], although it was found to further reduce RLUC activity at longer exposure times (unpublished data). Hence, the loss of RLUC activity could be explained by covalent modification of the protein that caused it to assume a non-native conformation incompatible with enzymatic activity. Depending on the extent of the conformational change induced by covalent modification, RLUC, and other proteins, may lose their aqueous solubility and aggregate. This was in fact observed in experiments in which cells were exposed to different concentrations of the compounds, and detergent-soluble and detergent-insoluble extract fractions were analyzed by Western blots probed with antibodies against RLUC and selected cellular proteins (Figure 2d). For some of the proteins tested, unfolding was substantial, with more than half of the protein amounts distributed to the detergent-insoluble fraction. IHSF058 appeared to cause somewhat less unfolding than WA and IHSF15, in agreement with the lower potency (i.e., the lower reactivity of its α,β-unsaturated carbonyl function) of the compound in the RLUC inactivation assay and its lower cytotoxicity.

Proteomic analysis revealed that in HeLa-derived cells exposed for 6 h to 12.5 µM WA, 915 of the 5132 proteins that were detected accumulated significantly (more than 1.5 fold) in the detergent-insoluble fraction (Table S1). When cells were exposed to 25 µM IHSF115 or IHSF058, 991 and 722 proteins, respectively, were found to unfold to some extent. Of the 915 proteins observed to unfold in WA-treated cells, 666 (73%) were also caused to aggregate in IHSF115-treated cells. WA and IHSF115 are structurally very different. The only element they appear to share is an α,β-unsaturated carbonyl moiety. Hence, a large majority of the denatured protein was shared between WA- and IHSF115-exposed cells, supporting our previous assertion that the compounds have little specific affinity for most proteins and largely act as electrophilic reactants. That a minority of proteins are induced to unfold by WA but not IHSF115 and vice versa suggests that steric effects limited the accessibility of some protein thiol groups to WA and of others to IHSF115, owing to the different structures of the compounds. Such steric hindrance may afford the compounds a small modicum of selectivity. One would expect this effect to be independent of compound concentration, except that aggregation may be more difficult to detect at low compound concentrations. It follows that observations made at higher compound concentrations are likely to provide a more comprehensive identification of proteins induced to unfold than observations made at lower concentrations and that findings made at higher concentrations should be relevant to what occurs at lower concentrations. In that sense, an observation that a target protein identified in the proteomic experiments of Dom et al. [124] in which cells were exposed to a WA concentration that was slightly below the EC50 value for loss of viability (1 µM at an EC50 value of 1.5 µM for MM1R cells) was susceptible to unfolding at a higher WA concentration (12.5 µM at an EC50 value for HeLa cells of 3.5 µM) is relevant because it demonstrates that modification of the protein by WA inactivates the protein. Thus, the protein is validated as a WA target of potential biological significance. Of the 209 identified direct targets of WA, 36 were found to unfold (Table S1; see also Table 3). The downregulation of proteins in WA-treated cells may be a consequence of their unfolding and aggregation. Four of the fifty-three downregulated proteins identified by Dom et al. [124] were found to aggregate in HeLa cells. Why is the correspondence between the two approaches for identifying WA targets so incomplete? An obvious reason may be that the covalent modification of a protein by WA may often not compromise its structural integrity. Depending on the location of the modification, a protein may or may not lose its activity or ability to normally interact with other proteins. Another reason (discussed further below) may be that different sets of proteins are targeted by WA in different cell types. The study that discovered WA-modified proteins [124] employed multiple myeloma cells, whereas the study that identified proteins caused to aggregate by WA [120] had been performed in cervical cancer cells. Finally, the studies may not have provided a complete accounting of WA-modified and aggregated proteins.

Proteins that aggregated in HeLa-derived cells treated with IHSF058 were compared with proteins that were bound by the compound in situ (employing a biotinylated probe) [120]. It was found that of the 722 proteins that were induced to aggregate by IHSF058 only 150 were bound to the IHSF058 probe. While it could not be excluded that covalent modification was incomplete (the probe possessing an arm that is not present in IHSF058), the result suggests that a large number of proteins were caused to unfold even though they were not themselves bound by IHSF058. These proteins may have become conformationally compromised due to an absence of necessary interactions with covalently modified and thereby disabled chaperones, subunits, or associating proteins. There is no reason why this finding should not also be relevant to WA. Hence, the analysis of proteins that were caused to unfold by WA may not only have discovered proteins that were directly targeted by WA but also a large group of proteins that unfolded because an interaction partner had been targeted by WA. It stands to reason that the latter not directly targeted proteins contributed as importantly to the biological effects of WA exposure as the directly targeted proteins.

**Figure 3:**
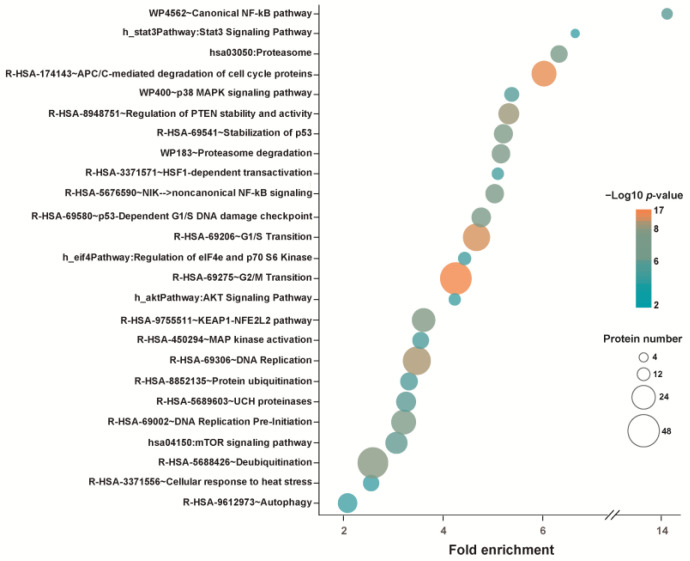
Selected results from a pathway analysis of proteins unfolded (accumulated in the detergent-insoluble fraction) in M1 cells after exposure to 12.5 µM WA. The underlying data were taken from ref. [120].

Selective results of functional enrichment analyses using DAVID bioinformatic resources [133], identifying pathways that may be impaired or inhibited because of protein unfolding induced by WA, are presented in Figure 3. Pathways related to cell cycle control such as “DNA replication”, “DNA replication pre-initiation”, “G1/S transition”, “p53-Dependent G1/S DNA damage checkpoint”, “G2/M Transition”, and “APC/C-mediated degradation of cell cycle proteins” were detected. Terms identified also include “autophagy”, “proteasome” and “proteasome degradation”, “protein ubiquitination” and “de-ubiquitination”, “UCH proteinases”, or “regulation of PTEN stability and activity” categories. Pathways related to “HSF1-dependent transactivation” and “cellular response to heat stress” were also found to be enriched. Categories affected by WA-induced aggregation include the “canonical NF-kB pathway” as well as “noncanonical NF-kB signaling”, “Stat3 signaling pathway”, “stabilization of p53”, “p38 MAPK signaling pathway” and “MAP kinase activation”, “AKT signaling pathway”, “KEAP1-NFE2L2 pathway”, “mTOR signaling pathway”, and “regulation of eIF4e and p70 S6 Kinase”. It is noted that, by its nature, this analysis was incapable of detecting targets of WA that were functionally but not structurally compromised, although it is not clear whether this represents a serious limitation. Furthermore, this analysis as well as the aforementioned studies that discovered proteins covalently modified by WA were aimed at identifying the particular pathways that were affected by WA. They ignored the possibility (discussed in Section 5) that the wholesale unfolding of proteins rather than the inactivation of a particular pathway by WA could be the immediate cause of cell death.

## 3. Proteomic Studies with CEL—Identification of Direct Targets of CEL

**Figure 4:**
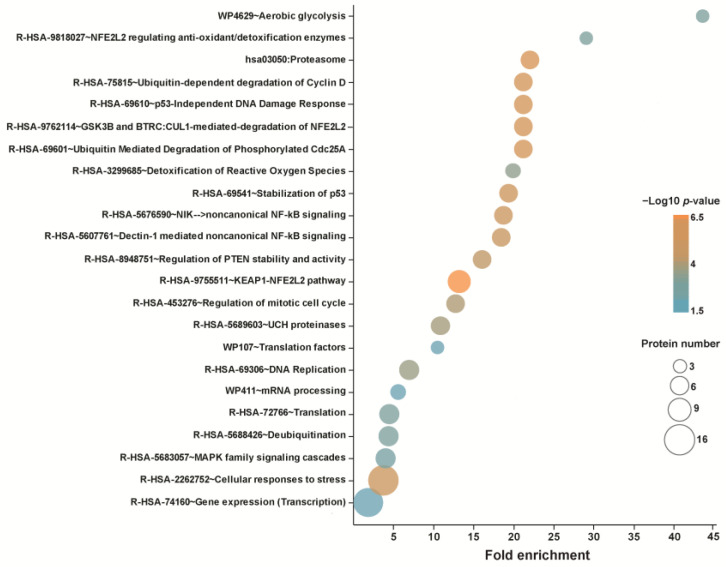
Selected results from a pathway analysis of direct CEL targets identified by Zhou et al. [122].

Zhou et al. [122] identified 66 direct targets of CEL in HeLa cells (listed in Table S2). A competitive labeling approach was used in which cells were first exposed or not exposed to CEL and then to the probe iodoacetamide-yne. Biotin was added to the probe by the click reaction using biotin-azide. After pulldown, proteins were subjected to on-bead tryptic digestion followed by mass spectrometry. A proteomic analysis of human colon cancer cells by Zhang et al. [134] revealed 100 direct CEL targets (listed in Table S2). The latter study employed a CEL probe prepared by the addition of an alkyne at a position far removed from the quinone methide moiety of CEL. Extracts from CEL-treated and control cells were processed using a protocol similar to that described in the Zhou et al. study. Selective results of a pathway/reactome analysis of the 66 CEL targets of the Zhou et al. study are shown in Figure 4. Targets revealed by this analysis included proteins related to proteasome-mediated processes, deubiquitination, the NRF2-controlled oxidative stress response, the heat shock response, DNA replication, transcription, translation, MAPK signaling, and aerobic glycolysis. A CEL probe was also used by Hong et al. [135] to identify 1157 CEL targets in mouse astrocytes. Pathways affected by the compound related to protein processing in the endoplasmic reticulum, ubiquitin-mediated proteolysis, and proteasome. Another study that employed a similar strategy uncovered 120 proteins bound by CEL in rat primary neurons [136].

**Figure 5:**
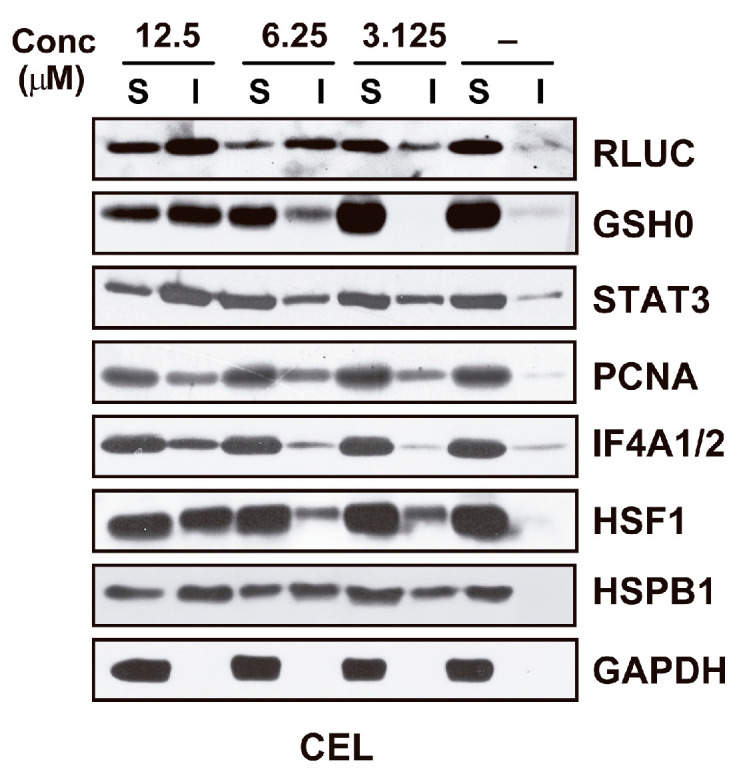
Western blot documenting the unfolding of selected proteins in M1 cells exposed for 6 h to vehicle (−) or CEL at the indicated concentrations. S: detergent-soluble extract fraction; I: detergent-insoluble extract fraction. The data shown were taken from ref. [120].

CEL was found to be highly active in the RLUC inactivation assay, and the nucleophile NAC partially neutralized this effect [120]. Western blots of detergent-soluble and detergent-insoluble fractions of CEL-treated cells probed with antibodies against selected proteins revealed substantial unfolding of these proteins (except GAPDH) (Figure 5). A proteomic analysis of proteins induced to aggregate by CEL was not performed.

## 4. The Cell Context Generates Targeting Specificity

Somewhat surprisingly, there is little overlap between the direct targets of CEL identified in the above-discussed studies by Zhou et al. [122] and Zhang et al. [134]. One of the studies identified 100 direct targets in colon cancer cells and the other 66 CEL targets in HeLa cells. Only 11 CEL targets were shared between the two studies (Table S2). This observation is in stark contrast with the results of the above-discussed comparison of proteins caused to unfold by WA and unrelated compound IHSF115 in HeLa-derived cells in which comparison revealed that the compounds modify proteins with little specificity. Barring technical reasons, the observation suggests that cellular background dominantly influences the probability with which proteins are modified by CEL. Kumar et al. [137] made analogous observations in a proteomic study of proteins upregulated or downregulated by WA in three prostate cancer cell lines. For one of these cell lines, LNCaP, 278 proteins were downregulated after 4 h of WA treatment, and 378 were downregulated after 24 h. Only six and twenty-one of these proteins, respectively, were also found downregulated in the other two cell lines. Similar findings were made for the other two lines. How could the cellular context provide targeting specificity? One explanation might be that many, perhaps nearly all, proteins are capable of engaging with other proteins, forming transient complexes that may shield their nucleophilic amino acid side chains from electrophilic attack. Proteomic differences between cell types, i.e., differences in relative amounts of proteins, may affect the probabilities of forming particular protective protein complexes as well as the persistence of these complexes. While the existence of extensive protein interactomes is well known, the latter observations suggest that they differ dramatically in different cell types. Despite these rationalizations, it remains astonishing that the cellular context imparts such a degree of specificity on WA and CEL, compounds (as discussed in Section 2) which appear to be little more than electrophilic reactants.

## 5. The Primary Mechanism of Action of WA and CEL Appears to Be the Induction of Proteotoxic Stress

**Figure 6:**
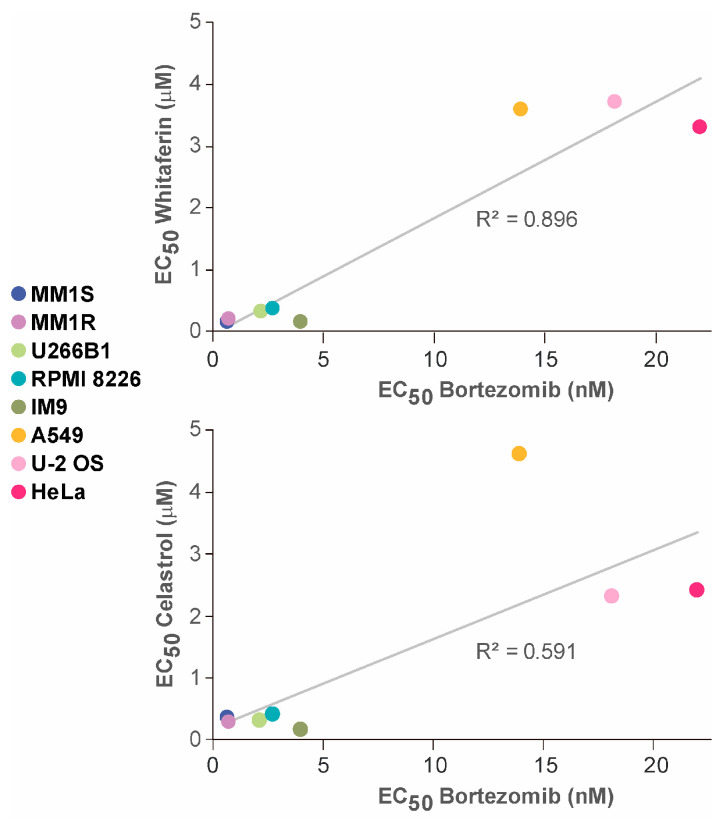
Correlation of the sensitivities of the indicated cell lines to WA (**top** graph) or CEL (**bottom** graph) and to bortezomib. Viabilities were estimated with an alamar blue assay. See the text for further details.

When cells are exposed to WA or CEL, large numbers of cellular proteins are covalently modified by the compounds. Some of these proteins lose activity and some, or perhaps most, partially unfold, some to a degree that results in their aggregation. Hence, an early consequence is an accumulation of non-native proteins. It, therefore, seemed reasonable to ask whether the primary mechanism by which compounds such as WA or CEL act is the generation of proteotoxic stress (as opposed to the inactivation of a particular protein or pathway). Specific proteasome inhibitors such as bortezomib are compounds that unquestionably exert their activity by inducing proteotoxic stress. Inhibition of the proteasome results in an accumulation of ubiquitinated and non-ubiquitinated non-native proteins. In experiments primarily aimed at demonstrating that IHSFs are particularly effective in killing multiple myeloma cells, EC50 values for loss of viability caused by treatment with IHSFs, WA, and CEL for 96 h were determined for sets of multiple myeloma and comparison cell lines [120]. To inquire whether the primary mechanism of cell death caused by WA and CEL might be the induction of proteotoxic stress, we asked whether the sensitivities to WA and CEL of the cancer cells employed in the latter study correlated with their sensitivities to bortezomib. Using EC50 data for bortezomib from “The Genomics of Drug Sensitivity in Cancer Project” (https://www.cancerrxgene.org/, accessed on 22 September 2023), we found a fair correlation for WA and a weaker correlation for CEL. The latter EC50 data for bortezomib were generated with cells incubated for 48 h in a standardized medium. When we employed our own EC50 data generated under the same experimental conditions that had been employed to determine sensitivity to WA and CEL [120], i.e., the same incubation time, specific culture media, and viability assay, we obtained a strong correlation of sensitivities for WA and bortezomib and a better correlation of sensitivities to CEL and bortezomib (Figure 6). Based on these correlations, we suggest that the primary mechanism of action of WA, and probably CEL as well, is the generation of proteotoxic stress. Although the compounds have been reported to inhibit proteasome activity, the question of whether the proteasome is directly inhibited by the compounds has remained controversial. However, previous studies showing that exposure to WA and CEL results in an accumulation of polyubiquitinated proteins lend further support to the hypothesis [122,124].

The reader may notice that EC50 values for WA and CEL were much higher than those for bortezomib. This large difference may be explained by the lack of affinity of WA and CEL for the proteins they target, whereas bortezomib specifically interacts with the proteasome.

Regarding possible mechanisms by which proteotoxic stress induced by WA or CEL may cause cell death, we thought it might be helpful to consider the mechanisms by which bortezomib has been proposed to cause cancer cell death. One such mechanism is based on the accumulation of non-native ubiquitinated and non-ubiquitinated proteins in the endoplasmic reticulum (ER) [138,139]. This results in the activation of the UPR and, if the ER stress is not neutralized, of the proapoptotic/terminal UPR, engaging protein kinase RNA-like endoplasmic reticulum kinase PERK, eukaryotic initiation factor eIF-2α, activating transcription factor 4 ATF4, and C/EBP homologous protein CHOP [38,105]. CHOP may enhance the expression of the proapoptotic protein PUMA (p53-upregulated modulator of apoptosis) [140]. Alternatively, prolonged UPR activity may cause apoptosis by a mechanism involving AP-1/JNK (activator protein 1/c-Jun N-terminal kinase) signaling [141]. Another mechanism is the inhibition of activation of NF-kB through the stabilization of inhibitor IkB [138], although questions remain to be answered [139]. Also, a study of six different multiple myeloma cell lines revealed that the cell lines responded differently to specific inhibition of NF-kB, suggesting that NF-kB inhibition may not be a generally effective mechanism of cell killing by proteotoxic stress [142]. Yet another suggested mechanism involves the stabilization of p53, BH3-interacting domain death agonist BID, and apoptosis regulator BAX, leading to apoptotic cell death [138]. The inhibition of autophagy through bortezomib-induced phosphorylation of extracellular signal-regulated kinase ERK has also been reported [143]. Concentrating on WA because of the particularly strong correlation of cell sensitivities to WA and bortezomib, it appears likely that the covalent modification of proteins in the ER results in the activation of the UPR and leads to UPR-mediated apoptotic cell death. WA is well known to cause ER stress and activate the UPR in human cancer cells [16,144] and even in *Xenopus laevis* kidney cells [145]. We noted that the proteomic study of Dom et al. [124] did not report the upregulation of ER stress-induced proteins (78 kDa glucose-regulated protein GRP78, X-box-binding protein 1 XBP1, endoplasmic reticulum-to-nucleus signaling 1 IRE1, activating transcription factor 6 ATF6, PERK, CHOP), perhaps because of the low WA concentration used in their experiments. The inhibition of NF-kB may also be a plausible mechanism of cell death (at least for the multiple myeloma cell line studied). Both components of NF-kB, p65 (RELA) and p50 (subunit 1, NFKB1), are direct targets of WA [124,126]. Subunits 1 and 2 (NFKB1 and NFKB2) as well as NEMO (NF-kappa-B essential modulator NEMO CC2-LZ domain-containing protein) and the IkB kinases IKKA and IKKB that are necessary for the activation of NF-kB by the canonical as well as noncanonical pathways, respectively, and other NF-kB pathway proteins are induced to unfold by WA [120]. WA may also interfere with autophagy. The compound covalently modifies several proteins involved in autophagy, including histone deacetylase 6 [126], and a multitude of autophagy-related proteins were found to aggregate (ref. [120], Table S1-6).

Like WA, CEL generates ER stress, leading to the activation of the UPR and, after prolonged activation of the UPR, to apoptotic cell death [105,106,146]. It may be reasonably expected that WA, CEL, and other compounds containing a reactive α,β-unsaturated carbonyl group will induce ER stress in any cell type and, in the absence of resolution of this stress, will cause UPR-mediated cell death.

## 6. Conclusions

Preparations made from the plants *Withania somnifera* and *Tripterygium wilfordii* Hook F have been used in traditional medicine for the treatment of a wide variety of medical conditions, including different types of cancers. Extensive work has shown that major bioactive compounds WA and CEL isolated from these plants recapitulate to a large degree the medicinal properties of unfractionated preparations. There has been considerable interest in the potential use of the compounds in cancer therapy. The latter studies have also led to the identification of multiple potential targets of the compounds, suggesting that they act by a mechanism that is not based on specific affinities for their targets. Both WA and CEL possess a reactive α,β-unsaturated carbonyl group (with WA additionally comprising a reactive epoxy group) that functions as a Michael acceptor of a nucleophile such as a thiol group of a protein. The fact that the cytotoxic activity of the compounds can be inhibited by nucleophilic reagent NAC confirms the latter notion that the compounds are reactants rather than drugs that interact with specific targets or affect specific processes. Proteomic studies identified large numbers (66–209 in the studies discussed herein) of proteins that are covalently modified by WA or CEL in different cell types, discovering multiple pathways that may potentially be affected by the compounds. For some proteins, the binding of WA or CEL may be without effect. The activity of other proteins may be impaired because WA or CEL binds within or close to their active site or to a region in which it disturbs the conformational integrity of the protein or blocks an interaction with a necessary partner protein. For some proteins, the binding of WA or CEL will have a sufficiently severe effect to result in significant unfolding and a concomitant loss of aqueous solubility. A proteomic study revealed that in cells exposed to a cytotoxic dose of WA, more than 900 proteins aggregated. Western blots showed that the extent of unfolding was substantial for some of these proteins. Nearly three-quarters of these proteins were also caused to unfold by IHSF115, a synthetic compound that shares no other similarity with WA than the presence of an α,β-unsaturated carbonyl group. This finding suggested that the binding of WA is essentially indiscriminate. Experiments with a related IHSF compound suggested that only a small minority of the unfolding proteins were covalently modified by the compound. This suggested that the proteostasis crisis caused by compounds such as WA or CEL was brought about by the unfolding not only of proteins directly targeted by the compounds but also of a greater number of proteins that had not interacted with the compounds and that, presumably, included proteins whose conformational integrity was maintained by interactions with proteins that were directly targeted. These findings shed a new light on the results of proteomic studies which identified proteins that are downregulated by WA or CEL. As discussed herein, one of the two studies discovered 66 CEL targets in HeLa cells, and the other identified 100 targets in colon cancer cells. Only 11 proteins were shared between the studies, and only 4 of the CEL targets were among the 209 WA targets identified in a multiple myeloma cell line. In a study of proteins downregulated in three different prostate cancer cell lines exposed to WA, only about 2% of the proteins downregulated in one of the cell lines (4 h exposure) were also downregulated in the other two cell lines. In light of the aforementioned study of protein aggregation suggesting that the compounds are largely non-discriminating, the apparent target specificity must have been generated by the different cell contexts. It seems reasonable to suggest that differences in the pervasive crosstalk of proteins in different cell types may explain these effects. The observation made with compound IHSF058 that most proteins induced to aggregate by compound exposure did not appear to be direct targets is also compatible with this notion of extensive interactions between proteins. Finally, regarding mechanisms of cell death, WA and CEL cause protein unfolding, i.e., generate proteotoxic stress in all cell compartments including the ER. Our demonstration that there is a tight correlation of the sensitivities of a set of cancer cell lines to WA and bortezomib, a specific inducer of proteotoxic stress, suggests that cells exposed to WA are killed as a result of proteotoxic stress. A fair correlation was also obtained for CEL. Exposure to bortezomib, WA, and CEL leads to the activation of the UPR which, upon prolonged activation, may cause apoptosis via the PERK/eIF-2/ATF4/CHOP pathway or another UPR-dependent pathway. Other mechanisms of cell death, as elaborated for the inhibition of NF-kB, may contribute in a cell type-specific fashion. As far as the potential use of WA and CEL in cancer therapy is concerned, it may be important to recognize that multiple myeloma cells are particularly sensitive to proteotoxic stress. Hence, there may be a therapeutic window for WA and CEL in the treatment of multiple myeloma that is similar to that for bortezomib and other proteasome inhibitors.

## Figures and Tables

**Table 1:** Targets of WA reported by individual studies.

Cancer Cell Line	Direct or Indirect Protein or Pathway Targets (Upregulated, Downregulated, or Post-Translationally Modified Proteins)	Refs.
U87, U251, GL26	Akt, mTOR, p70S6K, AMPKα, tuberin	[10]
MCF7, SUM159, SK-BR-3	WA binds to Cys303 of β-tubulin, α- and β-tubulin	[11]
MDA-MB-231, MCF7	FOXO3a, Bim	[12]
MDA-MB-231, MCF7	Complex III, Bax, Bak	[13]
MDA-MB-231, MCF7	Cdk1, Cdc25B/C	[14]
MDA-MB-231, MCF7	STAT3	[15]
MDA-MB-231, MCF7	Proteasome, autophagy	[16]
MDA-MB-231, MCF7, T-47D	ER-α, p53	[17]
MDA-MB-231, MCF7, T-47D, MDA-MB-68	p90-ribosomal S6 kinase, extracellular signal-regulated kinase 1/2	[18]
MDA-MB-231, MCF7, T-47D, MDA-MB-468	Autophagy	[19]
HCT116	STAT3	[20]
HCT-116, SW-480, SW-620	pS6K, p4E-BP1, Notch signaling	[21]
HCT116, SW480	Mad2, Cdc20	[22]
MDA-1986, JMAR, UM-SCC-2, JHU011	Cell cycle alteration	[23]
HepG2, SNU449	Keap1, Nrf2	[24]
H358, H460	ROS	[25]
H358, H460	Oxidative stress, lipid peroxidation, increased GSSG/GSH	[26]
A549	CAMs, Akt, NF-kB	[27]
A549	PI3K/Akt pathway	[28]
U-937	MMP, MAPK pathway	[29]
MDS92, MDS-L, HL-60, THP-1, Jurkat, Ramos	HMOX1, LC3A/B	[30]
MOLT-4, Jurkat, REH, K-562, HeLa, Saos-2, SP2/0	p38-MAPK signaling, ATF-2, HSP27	[31]
LY-10, LY-3, SudHL-6, Ramos, Raji, Mino, Jeko	Hsp90, NF-kB	[32]
HSC3, U-2 OS	mortalin-p53 interaction	[33]
PC-3, DU-145, LNCaP	c-Fos, HSPA6, Hsp70, c-FLIP(L)	[34]
Caki	STAT3	[35]
Caki	ROS, Bcl-2, Akt	[4]
M14, Mel501, SK28, Lu1205	Bcl-2, Bax	[36]

**Table 2:** Targets of CEL reported by individual studies.

Cancer Cell Type	Direct or Indirect Protein or Pathway Targets (Upregulated, Downregulated, or Post-Translationally Modified Proteins)	Refs.
Osteosarcoma cells	Activation of reactive oxygen species (ROS)/c-Jun N-terminal kinases (JNK) signaling	[81]
Gastric cancer cells	Downregulation of miR-21 expression; reduced phosphorylation of Akt, mTOR, and S6K and increased phosphorylation of AMPK	[82,83,84]
Androgen-receptor-positive prostate cancer cells	The AR/miR-101 axis; degradation of androgen receptor via HSP90 inhibition of calpain activation	[85,86,87]
Pancreatic cancer cells	Disruption of the HSP90-CDC37 interaction by targeting CDC37	[88,89]
HeLa (cervical cancer) cells and in vitro studies	Inhibition of p23	[90]
In vitro	Interaction with the carboxy-terminal region of HSP90A	[91]
Non-small-cell lung carcinoma, liver cancer, osteosarcoma, and hepatocellular carcinoma cells	Inhibition of mitochondrial respiratory chain (MRC) complex I and, consequently, ROS accumulation	[92,93,94,95]
Triple-negative breast cancer and melanoma cells	Mitochondrial dysfunction and PI3K/Akt/mTOR pathway inhibition	[96,97]
MCF-7 (breast cancer) cells	AMPK-dependent cell death; increased ROS levels; increased AMPK and p53 phosphorylation	[98]
Breast cancer cells	Destabilization of the ErbB2 and estrogen receptors	[99,100]
HL-60 (leukemia) cells	Inhibition of topoisomerase II	[101]
AML cells	Mitochondrial instability, activation of caspases, and downregulation of AML1-ETO/C-KIT oncoprotein, thus inhibiting the Akt, STAT3, and Erk1/2 downstream pathways	[102]
Hepatocellular carcinoma cells	Inhibition of STAT3/Janus kinase 2 (JAK2)	[103]
AML cells	Inhibition of Myb	[104]
Oral squamous cell carcinoma cells	Induction of unfolded protein response-dependent cell death, endoplasmic reticulum (ER) stress, and PERK-eukaryotic initiation factor 2 (eIF2)–activating transcription factor (ATF4)-C/EBP homology protein (CHOP) signaling	[105]
HeLa (cervical cancer) cells	Reduction in GSK3β levels	[106]
Glioblastoma cells	Inhibition of autophagy, accumulation of polyubiquitinated proteins, induction of the heat shock response, potentiating the heat shock response induced by HSP90 inhibition	[107]
Prostate cancer cells	Targeting of AR, ERG, and NF-kB signaling pathways	[108,109]
Gastric cancer cells	Upregulation of miR-146a expression, suppressing the NF-Kb activity	[110]
Multiple myeloma and prostate cancer cells; breast cancer xenografts	Inhibition of NF-kB	[111,112,113,114,115,116,117]
Prostate cancer cells	Downregulation of IL-6 gene expression via NF-kB inhibition	[118]
Prostate cancer xenografts and cells	Inhibition of the proteasome system	[114,115]

**Table 3:** Direct and indirect protein targets of WA.

Pathway	Direct WA Target or Downregulation by WA	Protein	UniProtKB Entry Name	Role/Function	Aggregation Observed
Oxidative stress (NRF2)	Target	Peroxiredoxin 1	PRDX1_HUMAN	Dual functioning thiol-specific peroxidase and molecular chaperon	No
	Target	Kelch-like ECH- associated protein 1	KEAP1_HUMAN	Inhibitor of NRF2	No
	Downregulation	Glutathione peroxidase 1	GPX1_HUMAN	Reduces hydrogen peroxide to water	No
	Downregulation	Phospholipid hydroperoxide glutathione peroxidase	GPX4_HUMAN	Reduces phospholipid hydroperoxide	No
Ubiquitin- proteasome system	Target	Proteasome subunit beta type-10	PSB10_HUMAN	Proteasome subunit involved in antigen processing	No
	Target	Proteasome subunit beta type-5	PSB5_HUMAN	Chymotrypsin-like activity of the 20S proteasome	No
	Target	AAA+ chaperone p97	TERA_HUMAN	Transitional endoplasmic reticulum ATPase: involved in the transport of ubiquitinated proteins and autophagy	No
	Target	Ubiquitin carboxyl- terminal hydrolase 14	UBP14_HUMAN	Deubiquitinating enzyme	No
	Target	Ubiquitin carboxyl- terminal hydrolase isozyme L5	UCHL5_HUMAN	Deubiquitinating enzyme	Yes
Autophagy	Target	Histone deacetylase 6	HDAC6_HUMAN	Deacetylation of lysine residues of histones and adapter protein	No
	Target	Phospholipase A-2- activating protein	PLAP_HUMAN	Autophagy cofactor	Yes
	Target	WD repeat domain phosphoinositide interacting protein 2	WIPI2_HUMAN	Formation of pre- autophagosome structures	Yes
	Target	SNARE-associated protein Snapin	SNAPN_HUMAN	Required for biogenesis of lysosome-related organelles	No
	Target Downregulation	Tubulin beta chain	TBB1_HUMAN	Major constituent of microtubules	No
	Downregulation	Annexin A4	ANXA4_HUMAN	Membrane fusion	No
Heat shock response	Target	Heat shock factor 1	HSF1_HUMAN	Master regulator of the heat shock response	Yes
	Target	Heat shock protein HSP 90-alpha	HS90A_HUMAN	Maturation and structural maintenance of proteins	No
	Target	Heat shock protein HSP 90-beta	HS90B_HUMAN	Maturation and structural maintenance of proteins	No
	Target	DnaJ homolog subfamily A member 2	DNJA2_HUMAN	Co-chaperone of HSP70 that stimulates ATP hydrolysis	No
	Target	BAG family molecular chaperone regulator 2	BAG2_HUMAN	Co-chaperone of HSP70	No
	Target	ER membrane protein complex subunit 9	EMC9_HUMAN	Chaperone complex that was relatively recently discovered	No
	Downregulation	Heat shock 70 kDa protein 13	HSP13_HUMAN	Processing of cytosolic and secretory proteins	No
	Downregulation	DnaJ homolog subfamily C member 10	DJC10_HUMAN	Disulfide reductase involved in the correct folding of proteins and degradation of misfolded proteins	No
	Downregulation	Peptidyl-prolyl cis-trans isomerase FKBP4	FKBP4_HUMAN	Prolyl isomerase and co-chaperone activities	No
Isomerase and disulfide reductase	Target	Peptidyl-prolyl cis-trans isomerase D	PPID_HUMAN	Co-chaperone of HSP90 complexes	Yes
	Target	Peptidyl-prolyl isomerase domain and WD repeat-containing protein 1	PPWD1_HUMAN	*cis-trans* isomerization of proline imidic peptide bonds	No
	Target	Peptidyl-prolyl cis-trans isomerase NIMA-interacting 1	PIN1_HUMAN	Phosphorylation-specific prolyl isomerase	No
	Downregulation	Peptidyl-prolyl cis-trans isomerase FKBP4	FKBP4_HUMAN	Prolyl isomerase and co-chaperone activities	No
	Downregulation	DnaJ homolog subfamily C member 10	DJC10_HUMAN	Disulfide reductase involved in the correct folding of proteins and degradation of misfolded proteins	No
ER- associated protein degradation	Target	ER degradation enhancing alpha- mannosidase-like protein 3	EDEM3_HUMAN	Catalyzes mannose trimming from Man8GlcNAc2 to Man7GlcNAc2	No
	Target	AAA+ chaperone p97 (VCP)	TERA_HUMAN	ATPase activity	No
	Target	Endoplasmic Reticulum-Golgi intermediate compartment protein 3	ERGI3_HUMAN	Transport between endoplasmic reticulum and Golgi	No
	Target	Homocysteine-responsive endoplasmic reticulum-resident ubiquitin-like domain member 1 protein	HERP1_HUMAN	Involved in ubiquitin- dependent degradation of misfolded endoplasmic reticulum proteins	No
	Target	Transport and Golgi organization protein 1 homolog	TGO1_HUMAN	Involved in the export process exported from the endoplasmic reticulum	No
Protein translation	Target	Eukaryotic translation initiation factor 5A-1	IF5A1_HUMAN	mRNA-binding protein involved in the level of mRNA turnover, acting downstream of decapping	Yes
	Target	Eukaryotic translation initiation factor 4B	IF4B_HUMAN	Required for the binding of mRNA to ribosomes	No
	Downregulation	Eukaryotic peptide chain release factor subunit 1	ERF1_HUMAN	Director of termination of the nascent peptide synthesis	No
	Downregulation	Eukaryotic translation initiation factor 3 subunit M	EIF3M_HUMAN	Responsible for the initiation of protein synthesis	No
Cytoskeleton functions	Target	Vimentin	VIME_HUMAN	An intermediate filament type III protein	No
	Target	Annexin A2	ANXA2_HUMAN	A multifunctional adapter protein that is part of the actin microfilament cytoskeleton	No
	Target	Tubulin beta	TUBB1_HUMAN	A major component of the microtubules	No
Cell cycle	Target	Dual specificity protein kinase TTK	TTK_HUMAN	Kinase essential for chromosome alignment	Yes
	Target	Nucleoporin Nup43	NUP43_HUMAN	Involved in kinetochore microtubule attachment, mitotic progression, and chromosome segregation	Yes
	Target	Protein phosphatase 1B	PPM1B_HUMAN	Phosphatase	No
	Target	Serrate RNA effector molecule homolog	SRRT_HUMAN	Mediator between the cap- binding complex (CBC) and the primary microRNAs (miRNAs) processing machinery during cell proliferation	No
	Target	Wings apart-like protein homolog	WAPL_HUMAN	Regulator of sister chromatid cohesion in mitosis	Yes
	Target	Nuclear-interacting partner of ALK	NIPA_HUMAN	Controls entering mitotic phase	No
	Target	Mini-chromosome maintenance complex-binding protein	MCMBP_HUMAN	Associated component of the MCM complex that acts as a regulator of DNA replication	Yes
	Target	Serine/threonine- protein phosphatase 2A 55 kDa regulatory subunit B alpha isoform	2ABA_HUMAN	Phosphatase. The B regulatory subunit modulates substrate selectivity and catalytic activity	No
Protein kinase functions	Target	TTK protein kinase	TTK_HUMAN	Involved in chromosome alignment	Yes
	Target	Serine/threonine- protein phosphatase 2A 65 kDa regulatory subunit A alpha isoform	2AAA_HUMAN	Phosphatase. The B regulatory subunit modulates substrate selectivity and catalytic activity	No
	Target	Serine/threonine- protein phosphatase 2A 55 kDa regulatory subunit B alpha isoform	2ABA_HUMAN	Phosphatase. The B regulatory subunit modulates substrate selectivity and catalytic activity	No
NFkB pathway	Target	Coiled-coil domain-containing protein 22	CCD22_HUMAN	Promoting IκB ubiquitination	Yes
	Target	Activating signal co- integrator 1 complex subunit 2	ASCC2_HUMAN	Transactivator of NFκB	No
	Target	ELKS/Rab6- interacting/CAST family member 1	RB6I2_HUMAN	Regulatory subunit of IKK	No
	Target	COMM domain- containing protein 3	COMD3_HUMAN	Downregulates activation of NFκB	Yes
	Target	Transcription factor p65 (RelA)	TF65_HUMAN	Transcription factor	Yes
	Target	Nuclear factor NFκB p105 subunit	NFκB1_HUMAN	DNA binding	No

## Supplementary Materials

The following supporting information can be downloaded at https://www.mdpi.com/article/10.3390/ijms25010367/s1.
