# Supplementary material for: Withaferin A and Celastrol Overwhelm Proteostasis
Source: Int J Mol Sci. 2023 Dec 27;25(1):367. doi: 10.3390/ijms25010367 (PMC10779417; doi:10.3390/ijms25010367)
Supplement: Supplementary file 1 [file ijms-25-00367-s001.zip › Contents of Supplementary Tables R1.pdf]

Table S1
Supplementary Table S1-1: Direct WA targets according to Dom et al. [124].
Supplementary Table S1-2: WA-downregulated proteins according to Dom et al. [124].
Supplementary Table S1-3: WA-downregulated proteins according to Narayan et al. [123].
Supplementary Table S1-4: WA-unfolded proteins according to Vilaboa et al. [120].
Supplementary Table S1-5: Direct WA targets of Dom et al. [124] that were found to aggregate.
Supplementary Table S1-6: WA-downregulated proteins of Dom et al. [124] that were found to aggregate.

Table S2
Supplementary Table S2-1: Direct CEL targets according to Zhou et al. [122].
Supplementary Table S2-2: Direct CEL targets according to Zhang et al. [134].
Supplementary Table S2-3: Direct CEL targets common to Zhou et al. [122] and Zhang et al. [134].
Supplementary Table S2-4: Direct WA targets of Dom et al. [124] that were also identified as direct CEL targets by Zhou et al. [122] and Zhang et al. 2022 [134].
